# Utility of Plant Growth-Promoting Rhizobacteria for Sustainable Production of Bermudagrass Forage

**DOI:** 10.3390/microorganisms11040863

**Published:** 2023-03-28

**Authors:** Kayla N. Sullins, S. Leanne Dillard, David W. Held, Elijah P. Carroll

**Affiliations:** 1 Department of Entomology and Plant Pathology, Auburn University, 301 Funchess Hall, Auburn, AL 36849, USA; kzs0070@auburn.edu (K.N.S.);; 2 Department of Animal Sciences, Auburn University, 210 Upchurch Hall, Auburn, AL 36849, USA

**Keywords:** sustainable agriculture, plant growth-promoting rhizobacteria (PGPR), bermudagrass, forage biomass, forage quality, mesofauna, biostimulant, biofertilization, soil health

## Abstract

A two-year study was conducted in bermudagrass hay fields in central Alabama to estimate the potential of plant growth-promoting rhizobacteria (PGPR) as a tool for sustainable agriculture in forage management. This study compared the effects of two treatments of PGPR, applied with and without lowered rates of nitrogen, to a full rate of nitrogen fertilizer in a hay production system. The PGPR treatments included a single-strain treatment of *Paenibacillus riograndensis* (DH44), and a blend including two *Bacillus pumilus* strains (AP7 and AP18) and a strain of *Bacillus sphaericus* (AP282). Data collection included estimates of forage biomass, forage quality, insect populations, soil mesofauna populations, and soil microbial respiration. Applications of PGPR with a half rate of fertilizer yielded similar forage biomass and quality results as that of a full rate of nitrogen. All PGPR treatments increased soil microbial respiration over time. Additionally, treatments containing *Paenibacillus riograndensis* positively influenced soil mesofauna populations. The results of this study indicated promising potential for PGPR applied with lowered nitrogen rates to reduce chemical inputs while maintaining yield and quality of forage.

## 1. Introduction

Grazinglands, grasslands, and rangelands cover an estimated 25–44% of Earth’s ice-free land surface [1,2], making grass ecosystems among the top single land uses on the planet. In the United States, the area devoted to forage, grasslands, and grazinglands is comparable to slightly more than forestland [3]. It comprises more than two-thirds of all agricultural land use [4]. Unlike other agricultural land uses in the United States, overall land use devoted to crops has decreased since 2007. However, lands devoted to grazing in the United States increased in area by 7% from 2007 to 2012 [3]. In the southeastern United States, bermudagrass (*Cynodon dactylon* and hybrids) is the most common warm-season forage grass planted on more than 12 million hectares. Bermudagrass, a drought-tolerant and persistent warm-season perennial grass, is popular because of its responsiveness to nitrogen fertilization and its high yield potential [5].

Nitrogen fertilization is key to productive forage systems, where multiple defoliation (above-ground biomass removal due to animal grazing, insect damage, or cutting for hay) events occur throughout the growing season. Nitrogen stimulates plant growth and increases the crude protein concentration of the forage [5], which improves overall nutritive value for livestock. Supplemental nitrogen fertilization of forages can also improve the quality and influence the feeding, abundance, or egg-laying behaviors of grass-feeding insects such as fall armyworms (*Spodoptera frugiperda*) or bermudagrass stem maggots (*Atherigona reversura*) [6]. Nitrogen fertilizer is the single most variable input cost in the production of forage grasses [7]. In the last five decades, the use of nitrogen, phosphorus, and potassium fertilizer has increased. Off-site movement of fertilizers and excess nutrient applications have led to issues such as soil salinity, heavy metal accumulation, water eutrophication, and accumulation of nitrates [8]. In grazed pastures, phosphorus runoff can cause water eutrophication in both coastal and freshwater systems [9]. Some coastal areas in the United States have already began implementing restrictions on fertilizer applications [10].

The application of nitrogen fertilizers can create microbial dead zones in the soil and decrease populations of mesofauna [11,12]. Soil microbes and mesofauna (i.e., soil mites and collembolans) that spend all or part of their lives in the soil [13] provide essential ecosystem services, such as decomposition of organic matter, nutrient cycling, and pest suppression [14]. Because fertilizers cannot be eliminated without drastic decreases in production, there is a need for integrated nutrient management that reduces the negative environmental impacts of fertilizers [15,16].

Plant-associated beneficial microbes can fix atmospheric nitrogen, solubilize pools of phosphorus in soils, and mitigate damage from grass-feeding pests or plant stress through tolerance or resistance mechanisms [17,18,19]. Plant growth-promoting rhizobacteria (PGPR) are free-living soil bacteria that can promote plant growth when applied as biostimulants and reduce the reliance on chemical fertilizers [15]. Previous studies conducted at Auburn University have identified several strains of endophytic PGPR that increase root and shoot growth in hybrid bermudagrasses [19,20]. After initial colonization, strains of PGPR can persist in bermudagrass roots and shoots for 8–12 weeks [21]. Thus far, these strains have only been evaluated in short-term greenhouse [22] or small field plot experiments [23]. The goal of this study was to evaluate PGPR in large forage plots at multiple sites over two years. We therefore selected a bermudagrass forage system to (1) compare the effects of PGPR and nitrogen fertilizers on forage biomass and quality, (2) compare the effects of PGPR and nitrogen fertilizers on insect populations, and (3) compare effects of PGPR and nitrogen fertilizers on soil mesofauna and soil microbial respiration.

## 2. Materials and Methods

### 2.1. Study Sites

A field experiment was conducted from May through October of 2020 and June through November of 2021 at two sites on the Edwin V. (E.V.) Smith Research Unit in Macon County, Alabama (32°25′26.7″ N 85°53′40.8″ W and 32°25′30.4″ N 85°53′48.7″ W). These sites had established stands of ‘Tifton 85’ bermudagrass utilized for hay production. The soil type at Site 1 was a Compass loamy sand with a taxonomic classification of coarse-loamy, siliceous, subactive, thermic Plinthic Paleudults, while Site 2 had a Luverne sandy loam taxonomically classified as fine, mixed, semiactive, thermic, Typic Hapludults. These soil types and classes were as specified by the National Resources Conservation Service (NRCS) web soil survey [24]. Variations in temperature and precipitation for the 2020 and 2021 field seasons were recorded as monthly averages (Supplementary Figure S1). Soil organic matter content averaged 2.0 and 1.9% at Sites 1 and 2, respectively. Fluctuations in soil temperature and soil moisture were also recorded (Supplementary Figure S2).

### 2.2. PGPR Strain Selection and Inoculate Production

A blend of three *Bacillus* strains, known as Blend 20, and a single-strain application of *Paenibacillus* sp. were used as PGPR inoculants. Blend 20 (B20) consists of two strains of *Bacillus pumilus* (AP7 and AP18), and a strain of *Bacillus sphaericus* (AP282). The single-strain application consisted of *Paenibacillus riograndensis* strain DH44 (DH44). These strains were selected from collections at Auburn University for their ability to increase growth promotion in bermudagrass based on previous studies [23]. Strains were grown as a bacterial lawn on TSA plates in the lab, then scraped into solution for field applications. All strains were grown in an incubator at 28 °C for their optimal growth dates. Optimal growth dates were determined based on concentration of spores when scraped into solution using sterile deionized water. AP282, *B. sphaericus*, was allowed to grow for three days before scraping. *B. pumilus* AP7 and AP18 were grown for 5 days, and *P. riograndensis* DH44 was grown for 10 to 12 days prior to scraping. Upon the optimal growth date, the bacteria were scraped into solution using autoclaved de-ionized water. After being scraped into 250 mL bottles, the PGPR were soaked in a water bath at 80 °C for 25 min to kill off the vegetative phase and leave endospores for field applications. Blend 20 and DH44 treatments were applied at a concentration of 5 × 10^6^ CFU/mL. 

### 2.3. Experimental Design, Treatments, and Field Evaluation

Plots, 3 m × 3 m, were arranged in an augmented factorial randomized block design with six treatments with three replicates per site. The six treatments included Blend 20 (B20), B20 plus a half rate of nitrogen fertilizer (B20 + N), DH44, DH44 plus a half rate of nitrogen fertilizer (DH44 + N), a full rate of nitrogen fertilizer (full-rate N), and a control plot (non-treated). Ammonium sulfate (21-0-0-24(S), Harrell’s, Lakeland, FL, USA) was used for nitrogen fertilizer applications. The full rate of nitrogen fertilizer applied was 112 kg N/ha and the half rate was 56 kg N/ha. These nitrogen rates were based on recommendations set forth by the Alabama Cooperative Extension System [25]. PGPR treatments were applied via foliar application using backpack sprayers at a rate of 500 mL per m^2^. Treatments were applied two times per year, with the initial application occurring in late May of 2020 and mid-June in 2021. The second application occurred 8 to 12 weeks after initial application (late July/early August in 2020 and late August in 2021).

Field samples were collected four times per year, with two harvests following each application. Harvests took place every 4 to 6 weeks after the initial application, with the second applications occurring immediately after the second harvest within the recommended practices for bermudagrass hay production [25]. Forage, insect, and mesofauna samples were collected every harvest for 2020 and 2021. Additionally, soil samples were collected at select harvests throughout year one and two to analyze other indicators of soil health.

### 2.4. Forage Sampling and Laboratory Analysis

Forage heights were recorded in field by averaging three random height measurements per plot. Forage biomass samples were collected by randomly placing six 0.3 m^2^ quadrats in each plot and cutting forage by hand to an approximate height of 5 cm using mechanical hedge trimmers (Makita, Makita U.S.A., Inc., La Mirada, CA, USA). Forage biomass clippings were then collected into individual bags and placed in a forced-air oven at 60 °C for 48 to 72 h. Dry weights were recorded as an estimate of forage biomass yield. Dried forage samples were then ground in a Wiley Mill (Thomas Scientific, Philadelphia, PA, USA) to pass through a 1 mm screen then sent to the University of Georgia Ag & Environmental Services Laboratories (Athens, GA, USA) for nutritive value analyses. Forage nutritive value metrics included crude protein (CP) percentage, neutral detergent fiber (NDF) percentage, acid detergent fiber (ADF) percentage, and total digestible nutrients (TDN). Forage analyses were conducted using near infrared spectroscopy (NIRS) using National Forage Testing Association certified prediction equations for bermudagrass.

### 2.5. Above-Ground Insect Sampling

Insect populations were sampled by taking 20 sweeps per plot with a standard insect sweep net. Samples were then transferred from the net into individual labeled bags and stored in a freezer until they were ready to be sorted and counted. Bermudagrass stem maggots (Diptera: Muscidae) and forage-feeding caterpillars, including armyworms (Lepidoptera: Noctuidae) and grass loopers (*Mocis* spp., Lepidoptera: Erebidae), were sorted and counted by hand. Other groups of grass-feeding insects counted include Acrididae, Tettigoniidae, Chrysomelidae, and Hemiptera.

### 2.6. Soil Sampling and Mesofauna Sampling

Soil mesofauna populations were estimated from soil cores collected in each plot using Par Aide HiO^TM^ hole cutters (1003-2, Par Aide, St. Paul, MN, USA). Two soil cores, 11.7 cm diameter, were taken from each plot, bagged into plastic bags, then placed into a cooler for transport to the lab [26]. In the lab, soil cores were placed in a Tullgren funnel system for 48 to 72 h under incandescent light to extract the soil arthropods [27]. Glass jars with a 70% ethanol solution were placed under each funnel to collect mesofauna as the soil dried. After the soil was completely dry, mesofauna samples were collected into 50 mL centrifuge tubes and stored in the refrigerator until they were ready to be counted. Soil mesofauna samples were counted under a dissecting microscope, focusing on Collembola, mesostigmatid, and oribatid mites as they are known indicators of soil health [14]. Voucher specimens of the morphotypes collected during this study are available through the Auburn University Biodiversity Learning Center, Auburn, AL, USA.

Additional soil samples were collected at harvest three during year one and harvest one, three, and four during year two to further analyze soil health. Soil samples from harvests one and three of both years were dried, sieved (No. 200), and weighed out to 100 g. These samples were then analyzed for soil microbial respiration at a commercial laboratory (Wade laboratories, Kearney, NE, USA). The procedure used a drying and re-wetting technique to collect the CO_2_–C ratio in ppm of C [28]. Soils collected at the final harvest of year two were boxed and sent to Auburn University Soil Testing Laboratory (Auburn, AL, USA) for organic matter (OM) content analysis. OM was determined by estimates using a loss-on-ignition method to measure organic carbon [29].

### 2.7. Statistical Analysis

Data were analyzed using PROC MIXED (SAS Institute Inc., Cary, NC, USA) for a completely randomized augmented factorial design. Orthogonal contrasts of PGPR treatments vs. controls (non-treated and full rate of nitrogen) were used for mean comparisons. Forage biomass, forage quality, insect populations, mesofauna populations, soil microbial respiration, and soil organic matter were analyzed separately. Site, year, and replicate were set as random variables and harvests were set as a repeated measure. Site and year were also analyzed separately to determine any individual effects from those parameters. PROC GLIMMIX with Tukey post hoc test (SAS Institute Inc., Cary, NC, USA) was used to compare main effects and interactions between years and sites. For forage biomass and quality, insect, and mesofauna populations, and soil microbial respiration, *p* ≤ 0.05 was used to determine significance.

## 3. Results

### 3.1. Forage Yield

**Figure 1:**
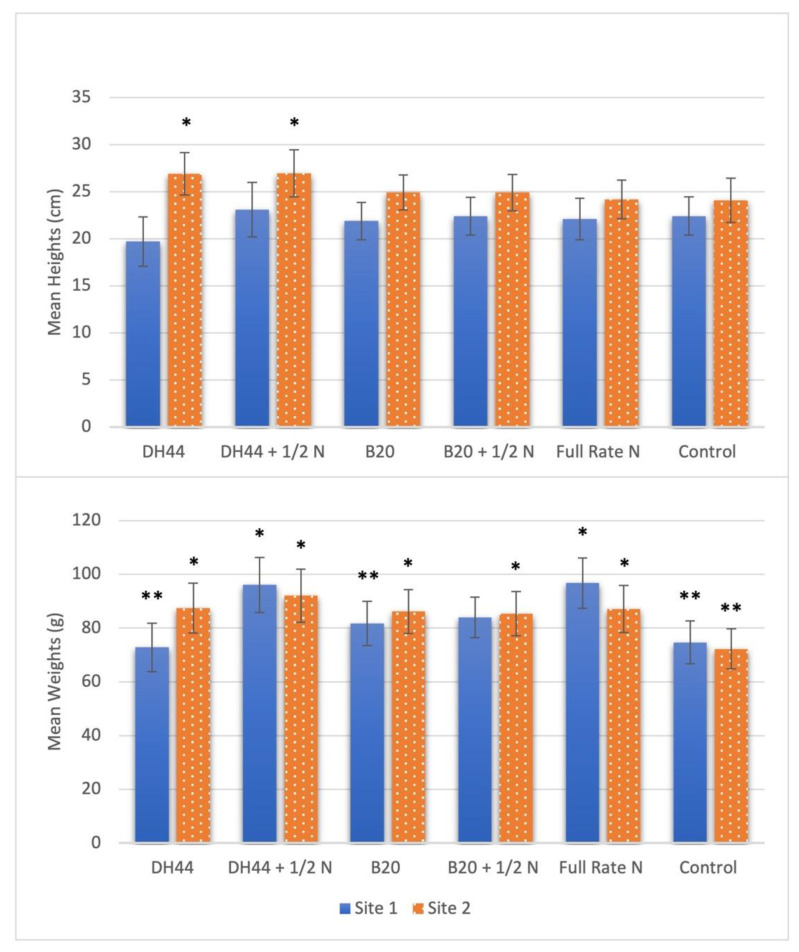
Mean forage yield (heights and weights) of bermudagrass plots harvested over two years from two sites in Alabama. Treatments included rhizobacterial inoculants (DH44 and B20) and those rhizobacteria applied with 56 kg N/ha (1/2 N). Treatments were compared to a full rate of nitrogen fertilizer (112 kg N/ha) as a positive control and a non-treated, negative control. “*” indicates significance from control (non-treated). (Orthogonal contrast, *p* ≤ 0.05). “**” indicates significance from full-rate N. (Orthogonal contrast, *p* ≤ 0.05).

Forage heights (Figure 1) throughout 2020 and 2021 averaged approximately 23.6 cm across all sites and plots and yielded no significant differences between treatments. However, significant differences between sites were observed (*p* < 0.001). At Site 1, the average height across all plots was 21.9 cm, with DH44 + N having the greatest mean and DH44 the least. No differences were observed between treatments at Site 1. At Site 2, the mean height across all treatments was 25.3 cm. The greatest mean height was observed in plots treated with DH44 + N followed by plots treated with DH44, which had similar heights. Mean heights at Site 2 were shortest in non-treated plots, which were shorter than plots treated with DH44 (*p* = 0.007) and DH44 + N (*p* = 0.016).

On both sites in 2020 and 2021, bermudagrass treated with DH44 + N yielded the greatest mean forage biomass on a dry matter basis and non-treated plots yielded the least (Figure 1). Plots treated with full-rate N (*p* = 0.012) and DH44 + N (*p* = 0.005) yielded greater biomass than non-treated plots regardless of year or site. In samples from 2021 across both sites, plots treated with DH44 yielded significantly greater amounts of forage biomass than non-treated plots (*p* = 0.007), but less than full-rate N plots (*p* = 0.032). These treatment differences were not observed in 2020. While significance differences between years were observed (*p* < 0.0001), no differences in forage dry weight were observed between sites (*p* = 0.49).

### 3.2. Forage Quality

Forage quality analysis consisted of percentages of crude protein (CP), acid-detergent fiber (ADF), neutral detergent fiber (NDF), and total digestible nutrients (TDN) from each forage sample (Table 1). Crude protein was greatest in plots treated with a full rate of nitrogen fertilizers and least in non-treated plots throughout 2020 and 2021 at both sites (*p* < 0.001). Additionally, B20 and DH44 treatments had significantly lower CP than plots treated with a full rate of N for both years (*p* < 0.001). During 2020, plots treated with either PGPR + N had similar CP to plots with the full rate of N (*p* ≥ 0.13). However, in 2021, plots treated with the full-rate N had significantly greater CP than plots treated with DH44 + N and B20 + N (*p* < 0.001).

Acid-detergent fiber was lowest in plots treated with a full rate of nitrogen and greatest in non-treated plots (*p* < 0.001). Across both sites in 2020 and 2021, B20 and DH44 treatments yielded significantly greater ADF than those of full-rate N plots (*p* < 0.001). Additionally, B20 + N and DH44 + N yielded significantly lower ADF than non-treated plots (*p* < 0.001). However, ADF differed by treatment between sites (*p* = 0.054). At Site 1, DH44 + N had greater ADF than full-rate N plots (*p* = 0.03) and DH44 plots had greater ADF than non-treated plots (*p* = 0.016). These treatment effects were not observed at Site 2. B20 + N yielded higher ADF than full-rate plots (*p* = 0.077) at Site 2, but not Site 1 (*p* = 0.815).

Neutral detergent fiber was lowest in plots treated with a full rate of nitrogen. These plots contained significantly lower NDF than B20 (*p* = 0.007), DH44 (*p =* 0.002), and non-treated plots (*p* = 0.004) across both sites and years. DH44 + N (*p* = 0.06) and B20 + N (*p* = 0.12) contained similar NDF as that of full-rate N. This interaction was not observed at Site 2 (*p* ≥ 0.314). Differences between years (*p* < 0.0001) and sites (*p* = 0.0085) were observed. During 2020, DH44 + N yielded higher NDF than full-rate N plots (*p* = 0.02), while during 2021 the treatments yielded similar percentages (*p* = 0.36). A similar trend was seen between these treatments at Site 1 (*p* = 0.02) and Site 2 (*p* = 0.31).

Total digestible nutrients (TDN) were greatest in plots treated with a full rate of nitrogen (52.7%). Throughout both sites in 2020 and 2021, B20 (*p* = 0.0088) and DH44 (*p* = 0.003) contained significantly lower TDN than those of plots treated with a full rate of nitrogen. TDN from plots treated with DH44 + N (*p* = 0.16) and B20 + N (*p* = 0.27) were similar to TDN from plots treated with full-rate N. No difference was observed between years (*p* = 0.38) and sites for TDN values (*p* = 0.29).

### 3.3. Above-Ground Insect Populations

An average of 73 ± 38.6 insects were recorded in sweep samples throughout this study. Plots treated with a full rate of nitrogen had the highest average with 80 ± 23.4 insects per sample, while non-treated plots averaged the lowest with 62 ± 17.9 insects per sample. Of those collected, 8% were bermudagrass stem maggots, *Atherigona reversura*, and <1% were forage-feeding caterpillars (e.g., *Spodoptera* spp. or *Mocis* spp.). Other insects counted in these samples include hemipterans (76%), grasshoppers (14%), and leaf beetles (1%). Hemipteran families were primarily represented by Cicadellidae, but also included small percentages of Cercopidae, Miridae, Lygaeidae, and Membracidae. Throughout both years of this study, plots treated with full-rate N had significantly greater numbers of acridid grasshoppers than non-treated plots (*p* = 0.028).

### 3.4. Soil Mesofauna Populations

Soil-dwelling mesofauna averaged 73 individuals per sample: 46% mesostigmatid mites, 41.8% oribatid mites, and 12.2% collembolans. The Mesostigmata mites collected were in the suborder Uropodina and the Oribatida mites collected were in the suborder Poronota. Families of collembolan collected were Oncopoduridae (48%), Entomobryidae (16%), Sminthuridae (14%), Isotomidae (14%), and Onychiuridae (8%).

Differences in mesostigmatid populations (Table 2) were observed between years (*p* < 0.001) and sites (*p* < 0.001). In 2020 and 2021, all plots treated with nitrogen fertilizer, including full-rate N (*p* < 0.001), DH44 + N (*p* < 0.001), and B20 + N (*p* = 0.001), contained significantly higher numbers of mesostigmatid mites than non-treated plots.

During 2020, plots treated with either PGPR (DH 44 or B20) + N had the highest numbers of mesostigmatids mites. DH44 + N (*p* = 0.006) and B20 +N (*p* = 0.009) had significantly greater counts than the non-treated plots. B20, DH44, and full-rate N plots all yielded similar counts and were not significantly different from each other (*p* ≥ 0.14). In 2021, the mesostigmatid counts in plots treated with a full rate of N yielded significantly more than all other plots in 2021, including DH44 (*p* = 0.013), DH44 + N (*p* = 0.024), B20 (*p* = 0.005), B20 + N (*p* = 0.012), and non-treated (*p* < 0.001). The non-treated plots had the lowest populations of mesostigmata and were significantly lower than DH44 (*p* = 0.032), DH44 + N (*p* = 0.017), and B20 + N (*p* = 0.033). At Site 1, there were significantly fewer mesostigmatids mites in plots treated with DH44 (*p* = 0.057) and B20 (*p* = 0.051) than in plots treated with full-rate N. These treatment differences were not observed at Site 2.

Oribatid mites (Table 3) on both sites in 2020 and 2021 were most abundant in plots treated with DH44 and lowest in non-treated plots. At both sites during 2020 and 2021, plots treated with DH44 (*p* = 0.003), DH44 + N (*p* = 0.061), B20 + N (*p* = 0.045), and full-rate N (*p* = 0.042) yielded greater oribatid counts than non-treated plots. During 2020, non-treated plots had significantly lower numbers of oribatid mites than plots treated with B20 + N (*p* = 0.048) and DH44 (*p* = 0.004). During 2021, oribatids were more abundant in plots treated with DH44 than non-treated (*p* = 0.074). No other significant treatment differences were observed in 2021. At Site 1, plots treated with B20 (*p* = 0.034) and B20 + N (*p* = 0.037) yielded significantly lower counts than plots treated with full-rate N. While at Site 2, DH44 plots yielded significantly higher counts than full-rate N (*p* = 0.015) or non-treated plots (*p* = 0.003).

Collembolans (Table 4) were lowest in plots treated with a full rate N fertilizer throughout this study (average of seven per sample). When both years were combined, plots treated with a full rate N fertilizer were significantly lower than DH44 (*p* = 0.009) and non-treated plots (*p* = 0.021). During 2020, non-treated plots had the highest collembolan counts, with an average of 27 collembola per sample. These counts were significantly greater than counts from all treated plots, including DH44 + N (*p* < 0.001), DH44 (*p* = 0.006), B20 + N (*p* < 0.001), B20 (*p* = 0.002), and full-rate N (*p* <0.001). DH44 plots were significantly greater than plots treated with full-rate N (*p* = 0.046). From 2020 to 2021, collembolan counts in all treated plots increased by an average of 272%, with DH44 (273%) and DH44 plus nitrogen (403%) having the highest increases. The highest collembolan counts for 2021 were observed in DH44 plots with an average of 58 collembola per sample. These plots yielded significantly greater numbers than those from plots treated with full-rate N (*p* = 0.028). In 2021, non-treated plots yielded similar counts as those treated with B20, B20 + N, and DH44 + N, with no significant difference between non-treated plots and these treatments (*p* > 0.05).

Treatment effects on collembolan counts also differed between sites (*p* = 0.0001) with no significant differences between treatments at Site 1. At Site 2, non-treated plots had the greatest number of collembolans per plot, while full-rate N plots had the lowest (*p* < 0.001). PGPR B20 (*p* = 0.018) and DH44 (*p* = 0.071) yielded higher counts than plots treated with full-rate N. Plots treated with DH44 + N (*p* < 0.001), B20 + N (*p* = 0.005), and DH44 (*p* = 0.024) yielded significantly lower counts than the non-treated plots.

### 3.5. Soil Microbial Respiration

**Figure 2:**
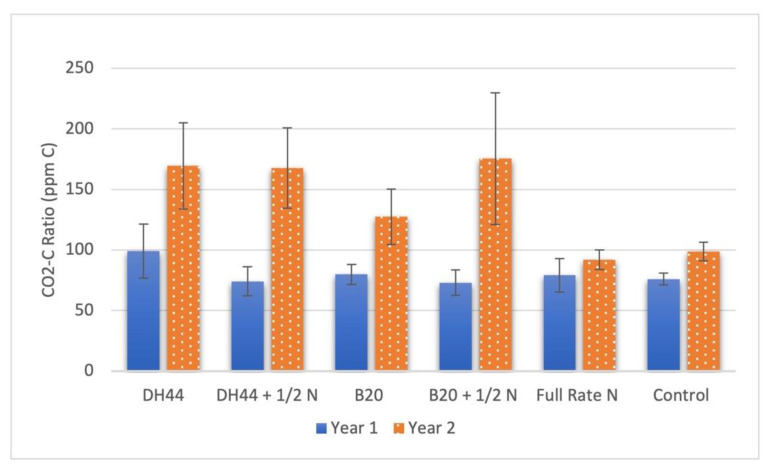
Mean soil microbial respiration (CO_2_-C ratio) in bermudagrass plots treated with conventional fertilizer or biofertilization. Treatments included rhizobacterial inoculants (DH44 and B20) and those rhizobacteria applied with 56 kg N/ha (1/2 N). Treatments were compared to a full rate of nitrogen fertilizer (112 kg N/ha) as a positive control and a non-treated, negative control. Respiration rates for PGPR plots in 2021 were significantly higher than in 2020. (*p* < 0.001).

Soil microbial respiration was greatest in plots treated with DH44 and B20 + N and lowest in plots treated with full-rate N (Figure 2). Full-rate N plots yielded significantly lower CO_2_-C ratios than that of plots treated with DH44 (*p* = 0.035) and B20 +N (*p* = 0.023). The soil microbial respiration increased in year 2 in plots treated with PGPR, but this increase was not observed for non-treated plots or those treated with the full rate of nitrogen. The largest increase (141%) was observed in plots treated with B20 + N, followed by a 127% increase in plots treated with DH44 + N. DH44 and B20 alone increased by 71% and 60% from year to year, respectively.

## 4. Discussion

This study evaluated the application of PGPR to large forage plots at multiple sites over two years. Based on our review of the literature, this is the first comprehensive study in forage production to compare forage biomass and quality, arthropod populations, and soil microbial respiration for two PGPR treatments applied as biostimulants, with conventional fertility using ammonium sulfate fertilizer.

In previous work from our labs [19,21,22,23], the benefits of adding PGPR, specifically DH44 and B20 alone or with a low rate of fertilizer, to grasses for enhanced growth or quality have been demonstrated in greenhouse and small plot field work. In this study, we used several common variables (dry mass, heights, CP, TDN, ADF, and NDF) to evaluate forage yield and quality following treatment. Because bermudagrass is very responsive to supplemental nitrogen fertilization [5], it is not surprising that ammonium sulfate at the full rate would produce high biomass and quality forage relative to non-treated plots. This also makes comparisons between conventional N sources of fertilizer and biological alternatives challenging. DH44 and DH44 + N, but not B20, can produce forage heights that were comparable to the full rate of N. Both DH44 and B20 caused growth promotion in greenhouse experiments [19,21] and the present field study suggests they can increase forage dry mass relative to untreated bermudagrass. However, only DH44 + N can produce dry mass yields similar to field application rates of ammonium sulfate. In previous work, B20 failed to produce similar forage dry mass as ammonium sulfate when applied during the growing season to potted bermudagrass in simulated field conditions [22]. However, B20 outperformed DH44 in dry mass yield when applied to small field plots in late summer to promote forage growth for stockpiling (standing hand production) [23]. These differences in strain performance may either be based on environmental factors or inherent differences in the ecology of these strains. DH44 and B20 strains differ in growth rates, origin, and performance. DH44 was isolated from bermudagrass growing in Alabama and each component of B20 was isolated from soils in crop fields in the midwestern United States [23].

For forage quality, plots treated with a full rate of nitrogen fertilizer produced significantly better quality. However, DH44 + N and B20 + N had similar percentages of CP, ADF, NDF, and TDN as grass from ammonium-sulfate-treated plots throughout most of this study. Both CP and TDN should be greater in higher quality forages, and higher ADF and NDF values indicate lower forage quality. Values for ADF are negatively correlated with digestibility, and NDF values are negatively correlated with forage intake by animals [5]. Full-rate N plots averaged CP of 13.76% and TDN of 52.69%, whereas DH44 or B20 with lower nitrogen rates had CP values of 11.7 to 13.1% and TDN of 50 to 52%. An average of 50% TDN is common for bermudagrass, and a lactating cow needs 10–12% CP [5]. When benchmarked against these standards, bermudagrass forage produced with applications of PGPR and reduced nitrogen rates would be adequate for livestock nutrition.

Improved forage quality under N fertilization also proved beneficial for grass-feeding insects such as grasshoppers. Throughout both years of this study, plots treated with the full rate of N had the highest average insect herbivores per sample and significantly higher numbers of acridid grasshoppers than non-treated plots. The PGPR or PGPR + N treatments did not appear to increase herbivore pressure. Previous work in our labs exposed caterpillars and white grubs to either topical application of PGPR or exposure to soil and grasses treated with PGPR [18,21] with no significant reduction in survival. In the case of grass-feeding caterpillars, the influence of PGPR applications to grass vary by the PGPR strains of blends. Blend 20, the PGPR blend used in this experiment, may negatively influence the oviposition behavior of moths in the field as noted in lab studies [18].

Apart from forage quality, this study also explored the potential agroecosystem benefits of microbial biofertilization. Grasslands are extensive global ecosystems and therefore a major contributor to ecosystems services and biogeochemical cycles. Soil microbial respiration is one such biogeochemical cycle that releases carbon into the atmosphere from terrestrial pools. Land management in grasslands, especially grazing, can decrease soil microbial respiration [30] and microbial biofertilizers have the potential to offset those reductions. In this study, soil microbial respiration was highest in plots treated with DH44 and B20 + N and lowest in plots treated with the full rate of fertilizer (Figure 2). Management practices in this study were applied equally to all plots, yet soil microbial respiration increased over time in plots treated with PGPR. This increase did not occur in non-treated plots or those treated with the full rate of synthetic fertilizer. These results further support the utility of microbial inoculants as biofertilizers to maintain or enhance carbon cycling in grasslands, especially those under management practices that may decrease soil microbial respiration.

Soil-dwelling mesofauna were maintained and/or increased in plots treated with DH44, but not for PGPR treatments in general. Mesostigmatid populations were highest in plots treated with nitrogen fertilizers, DH44 + N and the full rate of N. The literature suggests that mesostigmatid mites prefer lower soil pH [31]. This would explain the increase in populations in nitrogen plots throughout this study. Oribatid mites were abundant in all treated plots, including full rate of nitrogen plots. However, they were most abundant in plots treated with DH44 treatments [32]. Contrary to the trend in the literature [33,34], in this study collembolans were never more abundant in fertilized plots versus non-treated plots. However, two treatments (DH44 and DH44 + N) for year 2, Site 1 had nearly double the number of collembolans as in control plots. This was not significant by orthogonal contrasts but there were clearly outliers among all the plots. The mesofauna data indicate that certain PGPR strains have the potential to increase growth promotion while maintaining the soil biome to a degree that does not negatively impact populations of collembolans.

## 5. Conclusions

The extent and importance of grassland and rangeland ecosystems demands attention to management practices and inputs. Overall, the results of this study indicate that certain PGPR strains or blends applied alone or with reduced rates of nitrogen can provide comparable forage yield and quality to standard N inputs while positively impacting mesofauna populations and overall soil health. The study provides the first evidence that biological alternatives to nitrogen fertilizers, such as PGPR, can be a positive step towards sustainable forage production and sustainable agriculture.

## Figures and Tables

**Table 1:** Quality analysis for bermudagrass forage harvested from each site under conventional fertility or biofertilization.

Treatments	Mean ± SE No. Per Sample			
	CP	NDF	ADF	TDN
Site 1				
DH44	10.3 ± 0.24 ^N^	65.3 ± 0.72 ^N^	38.6 ± 0.42 ^N^*	49.6 ± 0.56 ^N^
DH44 ± N	11.7 ± 0.44 ^N^*	64.3 ± 0.78 ^N^	37.8 ± 0.47 ^N^*	50.4 ± 0.83 ^N^
B20	10.9 ± 0.32 ^N^	64.2 ± 0.71 ^N^	38.4 ± 0.58 ^N^	50.9 ± 0.71 ^N^
B20 ± N	12.4 ± 0.38 ^N^*	64.8 ± 0.69 ^N^	36.9 ± 0.44 *	51.4 ± 0.48
Full Rate of N	13.8 ± 0.46 *	62.7 ± 0.66 *	36.7 ± 0.49 *	52.3 ± 0.61
Control	10.7 ± 0.32 ^N^	65.5 ± 0.51 ^N^	39.8 ± 0.25 ^N^	50.7 ± 0.44
Site 2				
DH44	11.3 ± 0.34 ^N^	62.9 ± 1.1 ^N^	38.8 ± 0.46 ^N^	51 ± 0.79 ^N^
DH44 ± N	13.1 ± 0.49 *	61.7 ± 1.2	36.7 ± 0.58 *	52.3 ± 0.75
B20	11.8 ± 0.35 ^N^	63.2 ± 0.73 ^N^	39.2 ± 0.47 ^N^	50 ± 0.70 ^N^
B20 ± N	13.2 ± 0.55 *	60.8 ± 1.3	37.2 ± 0.66 ^N^*	52.8 ± 0.88
Full Rate of N	13.8 ± 0.56 *	60.4 ± 1.4	36.1 ± 0.73 *	53.1 ± 0.91 *
Control	11.5 ± 0.31 ^N^	62.5 ± 0.88	38.8 ± 0.44 ^N^	51 ± 0.71 ^N^

Treatments included rhizobacterial inoculants (DH44 and B20) and those rhizobacteria applied with 56 kg N/ha (1/2 N). Treatments were compared to a full rate of nitrogen fertilizer (112 kg N/ha) as a positive control and a non-treated, negative control. Crude protein (CP), acid-detergent fiber (ADF), neutral detergent fiber (NDF), and total digestible nutrients (TDN). “*” indicates significance from control (non-treated). (Orthogonal contrast, *p* ≤ 0.05). “^N^” indicates significance from full-rate N. (Orthogonal contrast— *p* ≤ 0.05).

**Table 2:** Abundance of mesostigmatids mites collected from bermudagrass forage plots treated with conventional fertility or biofertilization.

Treatments	Mean (±SEM) No. Per Sample			
	Year 1		Year 2	
	Site 1	Site 2	Site 1	Site 2
DH44	84.75 ± 13.49	101.9 ± 20.16	92.58 ± 19.27 ^N^	128.2 ± 20
DH44 ± N	132.3 ± 20.73 *	100 ± 19.49	92.67 ± 21.21 ^N^	140.7 ± 21.09 *
B20	95.42 ± 18.8	61.5 ± 16.65	79.83 ± 15.21 ^N^	123.8 ± 25.01
B20 ± N	138.2 ± 41.12 *	86.25 ± 17.07	54.17 ± 12.32 ^N^	165.8 ± 34.36 *
Full Rate of N	78.5 ± 12.26	78.5 ± 26.78	187.8 ± 34.49 *	159.9 ± 45.01 *
Control	47.92 ± 14.15	65.58 ± 15.58	50.92 ± 9.61 ^N^	60.83 ± 15.3 ^N^

Treatments included rhizobacterial inoculants (DH44 and B20) and those rhizobacteria applied with 56 kg N/ha (1/2 N). Treatments were compared to a full rate of nitrogen fertilizer (112 kg N/ha) as a positive control and a non-treated, negative control. “*” indicates significance from control (non-treated). (Orthogonal contrast, *p* ≤ 0.05). “^N^” indicates significance from full-rate N. (Orthogonal contrast, *p* ≤ 0.05).

**Table 3:** Abundance of oribatid mites collected from bermudagrass forage plots treated with conventional fertility or biofertilization.

Treatments	Mean (±SEM) No. Per Sample			
	Year 1		Year 2	
	Site 1	Site 2	Site 1	Site 2
DH44	69.67 ± 12.84	150.9 ± 40.2 ^N^*	63.42 ± 7.42	187.5 ± 65.3 *
DH44 ± N	93.83 ± 21.46 *	63.42 ± 16.27	71.5 ± 12.96	150.7 ± 33.32
B20	73.75 ± 14.74	94.17 ± 35.02	47.17 ± 7.64 ^N^	108.9 ± 29.17
B20 ± N	78.25 ± 21.18	103 ± 23.8	44 ± 6.84 ^N^	164.5 ± 47.47
Full Rate of N	82.33 ± 20.41	81.67 ± 15.78	128.5 ± 53.23 *	99.58 ± 30.26 *
Control	42.75 ± 9.64	58.42 ± 16.05	41.75 ± 7.58 ^N^	89.42 ± 24.4 ^N^

Treatments included rhizobacterial inoculants (DH44 and B20) and those rhizobacteria applied with 56 kg N/ha (1/2 N). Treatments were compared to a full rate of nitrogen fertilizer (112 kg N/ha) as a positive control and a non-treated, negative control. “*” indicates significance from control (non-treated). (Orthogonal contrast, *p* ≤ 0.05). “^N^” indicates significance from full-rate N. (Orthogonal contrast, *p* ≤ 0.05).

**Table 4:** Abundance of Collembola collected from bermudagrass forage plots treated with conventional fertility or biofertilization.

Treatments	Mean (±SEM) No. Per Sample			
	Year 1		Year 2	
	Site 1	Site 2	Site 1	Site 2
DH44	11 ± 3.97 *	19.92 ± 4.51	92.25 ± 37.01 ^N^	23.00 ± 4.89
DH44 ± N	7 ± 2.16 *	9.25 ± 2.36 *	70.58 ± 24.04	11.08 ± 3.6 *
B20	9.25 ± 2.86 *	18.00 ± 6.53	41.25 ± 10.09	32.00 ± 11.9 ^N^
B20 ± N	8.5 ± 4.31 *	12.50 ± 2.59 *	52.42 ± 17.61	23.33 ± 7.95
Full Rate of N	4.67 ± 1.53 *	9.17 ± 3.35 *	37.67 ± 8.69	11.33 ± 2.47 *
Control	23.08 ± 3.7 ^N^	31.58 ± 10 ^N^	42.92 ± 12.53	39.42 ± 8.98 ^N^

Treatments included rhizobacterial inoculants (DH44 and B20) and those rhizobacteria applied with 56 kg N/ha (1/2 N). Treatments were compared to a full rate of nitrogen fertilizer (112 kg N/ha) as a positive control and a non-treated, negative control. “*” indicates significance from control (non-treated). (Orthogonal contrast, *p* ≤ 0.05). “^N^” indicates significance from full-rate N. (Orthogonal contrast, *p* ≤ 0.05).

## Data Availability

The data are available upon request from the corresponding author.

## Supplementary Materials

The following supporting information can be downloaded at: https://www.mdpi.com/article/10.3390/microorganisms11040863/s1, Figure S1: Weather data in 2020 and 2021 at the E.V. Smith Research Station in Macon County, Alabama, USA; Figure S2: Soil conditions at 10 cm during the study in 2020 and 2021 at the E.V. Smith Research Station in Macon County, Alabama, USA.
