# Supplementary material for: Utility of Plant Growth-Promoting Rhizobacteria for Sustainable Production of Bermudagrass Forage
Source: Microorganisms. 2023 Mar 28;11(4):863. doi: 10.3390/microorganisms11040863 (PMC10144749; doi:10.3390/microorganisms11040863)
Supplement: Supplementary file 1 [file microorganisms-11-00863-s001.zip › microorganisms-2102547-supplementary.pdf]

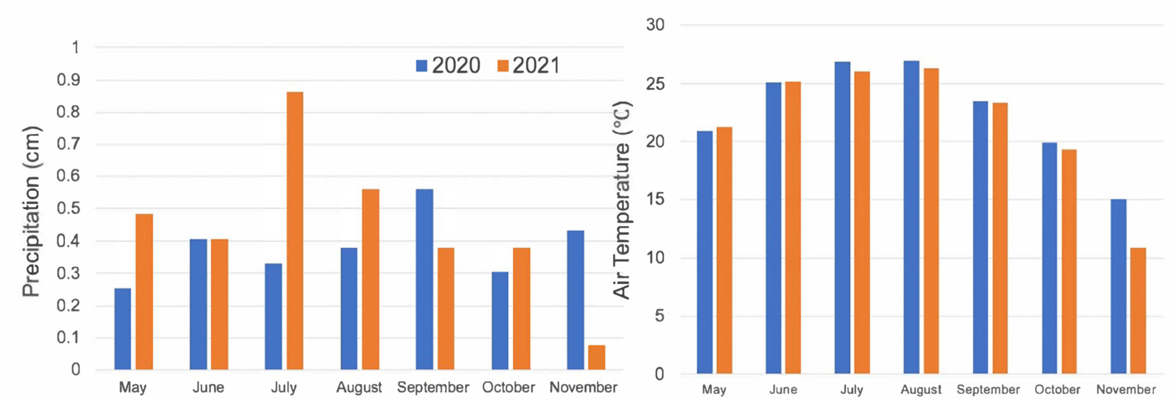

Supplemental Figure S1. Weather data during the study in 2020 and 2021 where PGPR stains and blends were applied with or without nitrogen to field plots of bermudagrass forage at E.V. Smith Research Station in Macon County, Alabama. The left figure is average monthly precipitation (cm) and average monthly air temperature (℃) is on the right.

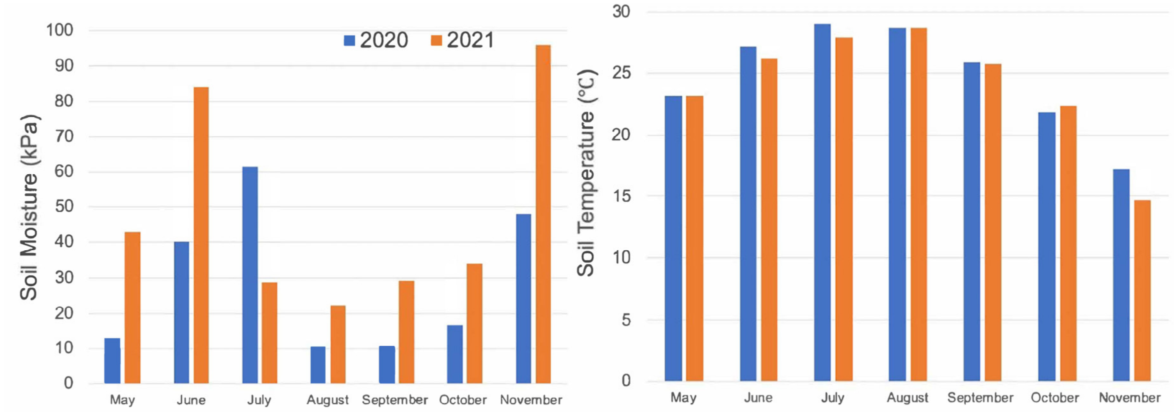

Supplemental Figure S2. Soil conditions at 10 cm during the study in 2020 and 2021 where PGPR stains and blends were applied with or without nitrogen to field plots of bermudagrass forage at E.V. Smith Research Station in Macon County, Alabama. The left figure is average monthly soil moisture (kPa) and average monthly soil temperature (℃) is on the right.
